# Meningioma growth beneath the outer membrane of a traumatic chronic subdural hematoma after burr-hole drainage: a case report and literature review

**DOI:** 10.3389/fonc.2025.1517778

**Published:** 2025-05-16

**Authors:** Tao Xiong, Wei-Xian Liu, Zhu-Xiao Tang, Jiang-Chun Ma, Hu Sun

**Affiliations:** Department of Neurosurgery, Brain Center, Zhejiang Hospital, Hangzhou, Zhejiang, China

**Keywords:** burr-hole drainage, chronic subdural hematomas, head trauma, inflammatory stimulation, meningiomas, outer membrane, postoperative

## Abstract

**Introduction:**

Growth of meningiomas secondary to postoperative chronic subdural hematoma is extremely rare. Here, we present the first report of a patient who developed a meningioma within the outer membrane of the chronic subdural hematoma after burr-hole drainage for a traumatic chronic subdural hematoma.

**Case presentation:**

A 75-year-old man underwent burr-hole drainage for a traumatic chronic subdural hematoma on the left side three years prior to presentation. Postoperative follow-up computed tomography revealed no recurrence of chronic subdural hematoma. The patient was admitted because of dizziness and immediately underwent magnetic resonance imaging (MRI), which also showed no recurrence of the chronic subdural hematoma; however, an abnormal signal lesion was identified in the left frontal region. Consequently, an enhanced MRI examination was performed, which indicated significant contrast enhancement, suggesting the diagnosis of meningioma. Subsequently, a frontotemporal craniotomy was performed, and the pathological diagnosis confirmed a meningioma (meningothelial type, World Health Organization grade I). Interestingly, during the craniotomy, the meningioma grew under the outer membrane of the chronic subdural hematoma, fused with the membrane, adhered tightly, and could not be separated. Fifteen months postoperatively, the patient was in good condition with no tumor recurrence.

**Conclusions:**

Meningioma growth beneath the outer membrane of traumatic chronic subdural hematoma following burr-hole drainage has not been previously reported, which further highlights the probable significant role of trauma and chronic subdural hematoma-induced inflammatory stimulation in meningioma occurrence and development.

## 1 Introduction

Chronic subdural hematoma (CSDH) is one of the most common diseases in neurosurgery.Especially in the elderly (1, 2).The formation and progression process of CSDH remains poorly understood. Possible explanatory hypotheses include minor head injury, inflammatory response, and transformation from acute subdural hematoma (2).Meningiomas are the most common intracranial tumors (3). And there may be a potential association between its progression and head injury(23, 24).But the reports of the relationship between meningiomas and chronic subdural hematomas (CSDH) are rare. The few available studies primarily observed cases of meningiomas associated with CSDH and mainly discusses the mechanism of intracranial hemorrhage caused by meningioma (4–8). Postoperative recurrence of CSDH is a common and concerning issue; however, postoperative complications of meningioma growth have rarely been reported. Here, we report a case of meningioma growth in the surgical area after burr-hole drainage of a traumatic CSDH, which was closely related to the outer membrane of the CSDH.

## 2 Case presentation

**Figure 1:**
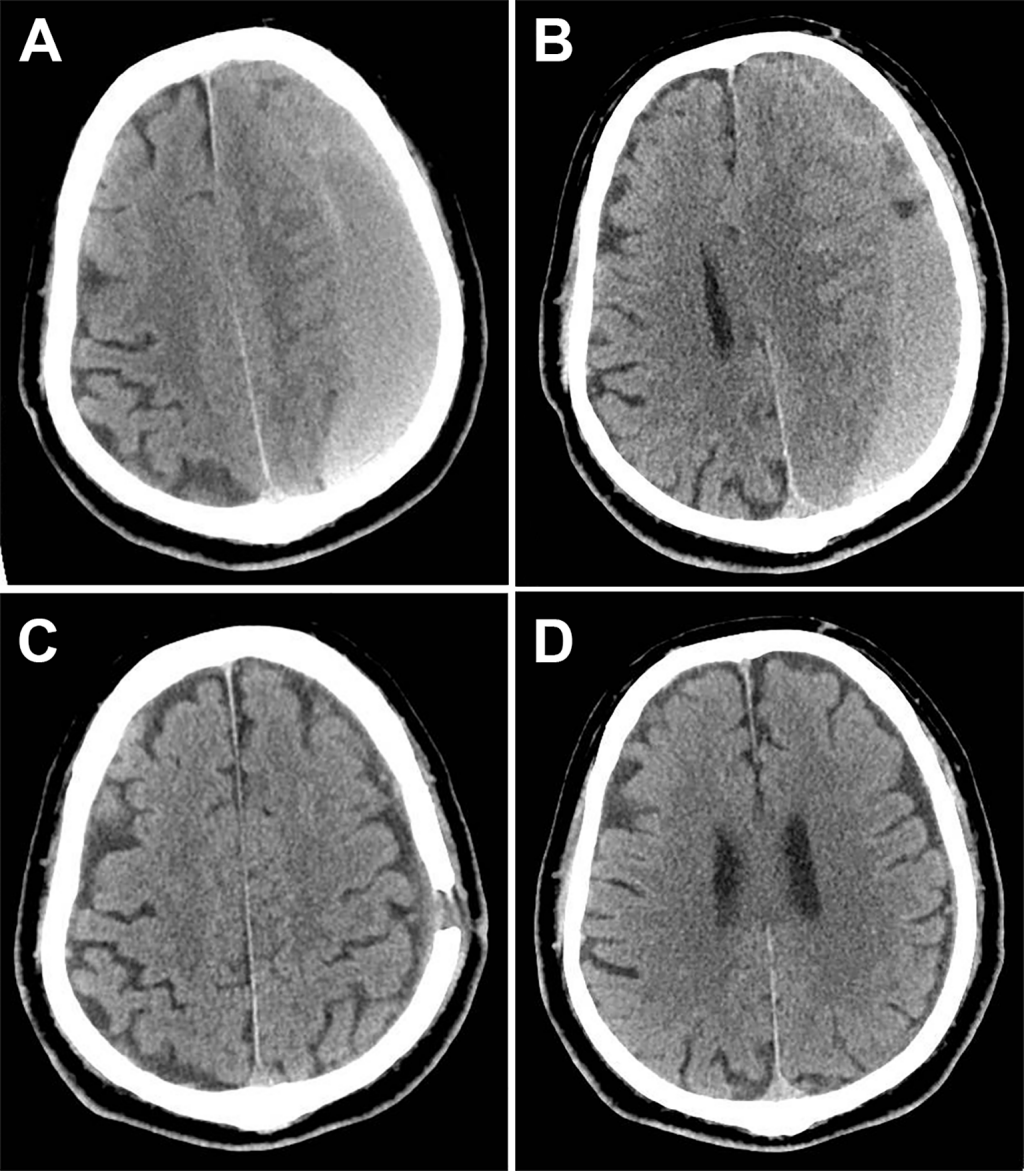
Computed tomography (CT) of the traumatic chronic subdural hematomas (CSDH) before and after surgery. **(A)** Preoperative CT showed hematoma thicker than 2 cm (1 month after head trauma). **(B)** Another layer indicates that the left lateral ventricle is completely compressed. **(C)** Six weeks after burr-hole drainage, CT showed no sign of recurrence. **(D)** The CT during the same time revealed no tumor or other abnormal lesions.

**Figure 2:**
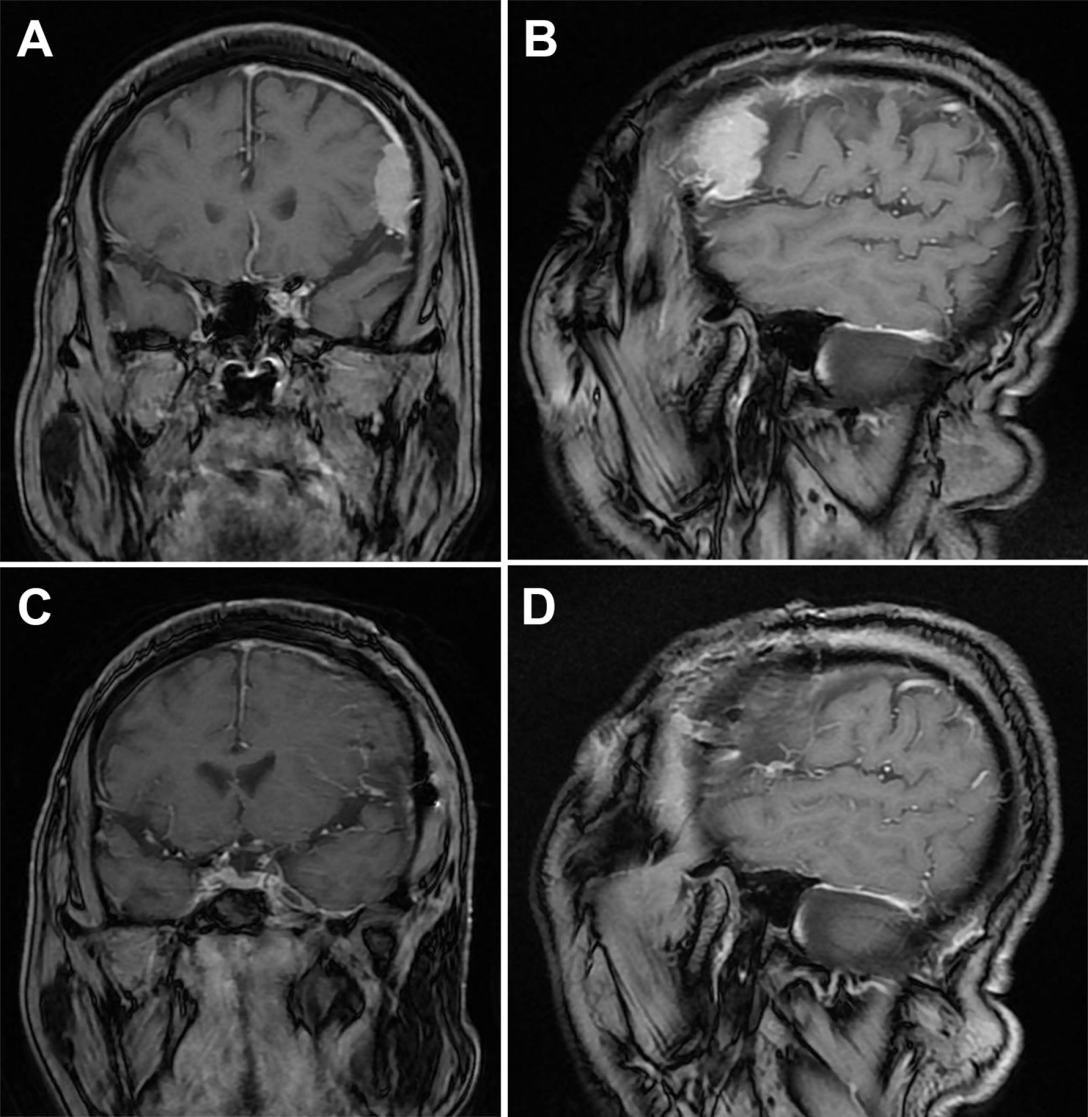
Magnetic resonance imaging (MRI) of meningioma Before and after surgery. **(A)** Coronal MRI at the largest level of the tumor. **(B)** Sagittal MRI at the largest level of the tumor (3.3×3×1.1 cm³). **(C)** Coronal MRI after craniotomy. **(D)** Sagittal MRI after craniotomy.

A 75-year-old male patient presented with dizziness for 5 days. Physical examination: The patient was alert and oriented. Bilateral pupils were equal in size and shape, measuring 3 mm in diameter, with brisk light reflexes. The scalp incision demonstrated excellent healing. Muscle strength and tone were within normal limits in all four extremities. No Babinski sign was elicited bilaterally.Since he had a history of burr-hole irrigation drainage for traumatic CSDH three years ago (**Figure 1**), a plain head MRI was initially performed, followed by an enhanced MRI to check the detected abnormality, which showed a markedly enhanced lesion in the left frontal region within the previous CSDH site, suggesting meningioma and no recurrence of CSDH (**Figure 2**). As the tumor was larger than 3 cm and the patient and his family strongly desired surgery, a left frontotemporal craniotomy for tumor resection was successfully performed, achieving Simpson grade 1 meningioma resection.

**Figure 3:**
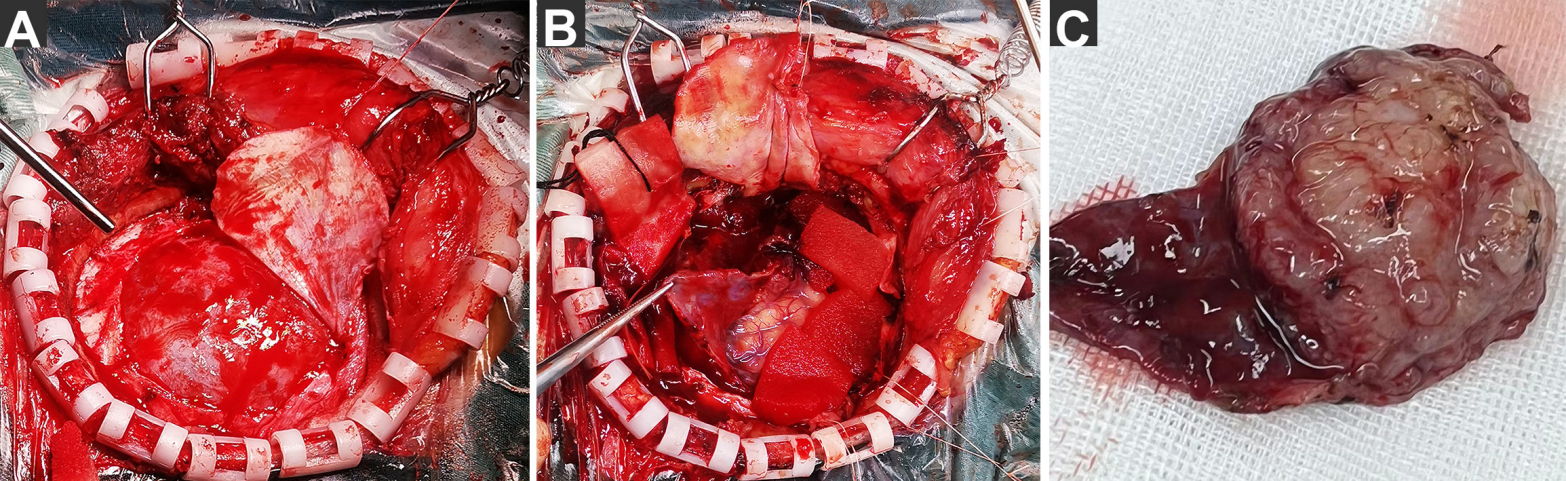
Photographs of extraordinary findings intraoperative. **(A)** After cutting through the dura mater, we found that the dura mater was intact and uninvaded. and a layer of membranous tissue was discovered covering the brain surface. **(B)** After the membrane was incised, a solid tumor was exposed, creeping growth along the membrane and fusing together. **(C)** The tumor was completely removed along with its surface membrane.

The findings during surgery were unusual (**Figure 3**); after cutting through the dura mater, we found that it was intact and uninvaded. A membranous tissue layer was discovered covering the brain surface, appearing reddish-gray and well-perfused, adhering to the inner dura mater, and extending in all directions without a clear end. Therefore, we realized that this was the external membrane of the CSDH. After the membrane was incised, we exposed a solid grayish-white tumor with moderate texture, creeping growth along the membrane, and fusing together form tumor base—the main source of blood supply. The membrane tissue was separated from the dura to the lateral side of the tumor border and excised. The tumor adhesion to the brain tissue was carefully separated at the arachnoid interface under a microscope and completely removed along with its surface membrane.

**Figure 4:**
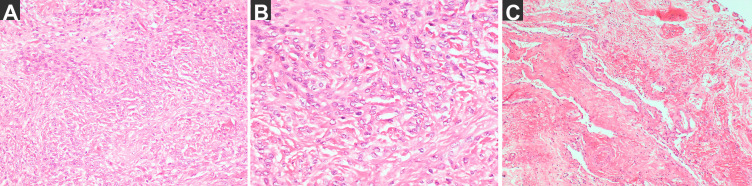
The pathological diagnosis. **(A)** the tumor H–E staining: meningioma, Meningothelial type, WHO grade I (magnification: 200×) and immunohistochemical staining results: SSTR (2 +), E-cadherin (membrane+), D2-40 (+), S-100 (nerve +), CK (Pan) (−), Vimentin (+), the EMA (local +), GFAP (−) and Ki67 (+ 2%), ER (−). **(B)** the tumor H–E staining (magnification: 400×). **(C)** The membrane: small pieces of fibrous cyst wall-like tissue with small vascular proliferation and slight inflammatory cell infiltration (magnification: 200×).

The pathological diagnosis (**Figure 4**) revealed a meningioma (meningothelial type, World Health Organization grade I), and the membrane consisted of small pieces of fibrous cyst wall-like tissue with small vascular proliferation and a slight amount of inflammatory cell infiltration.

## 3 Discussion

Meningiomas, usually solid and benign, have a prolonged disease course and slow progression. Therefore, the combination of meningiomas and intracranial hematomas is relatively infrequent and generally presents as an intratumoral, intraventricular, or subarachnoid hemorrhage (1). CSDH is a chronic space-occupying lesion characterized by the accumulation of blood and its degradation products, which form an encapsulated collection between the arachnoid and dura mater; its formation is related to head trauma and inflammatory responses (2, 9). Reports on the relationship between meningiomas and CSDH are scarce. In the few available studies, CSDH-associated meningiomas were primarily observed, and the mechanism of intracranial hemorrhage caused by meningioma has been reported (4–8). In contrast, in this case, the meningioma was found 3 years after surgically removing the traumatic CSDH, and grew under the external membrane of the CSDH and was closely related to it. Meningiomas usually have a wide base attachment to the dura mater because they originate from the cap cells of the arachnoid granules, and even break through the dura mater and destroy the skull (10, 11). Interestingly, during this surgery, the dura mater was intact with no signs of invasion. After the dura mater was incised and lifted, the outer membranous structure of the chronic subdural hematoma was first observed instead of the tumor, which became visible only after the membrane was carefully incised and lifted. The meningioma grew together with the membrane, and there was no obvious boundary between them. The membrane was rich in blood supply and thickened, much thicker than the outer membrane of a general CSDH. Blood supply of the tumor primarily came from this membrane tissue, and pathological examination of this membrane showed no tumor cells, but revealed angiogenic responses and inflammatory cell infiltration.

CSDH cavity and external membrane express high levels of HIF-1, VEGF, COX-2, Ang2, MMP, and other inflammatory cytokines (9, 12, 13). The association between VEGF and COX-2 with meningioma growth and proliferation is well known (12). VEGF plays an important role in the angiogenesis of meningiomas by promoting blood vessel formation, tumor growth, and malignant proliferation (14, 15). COX-2 primarily exerts its tumor-promoting effects by inhibiting tumor cell apoptosis and promoting new blood vessel formation in tumor tissues, with a synergistic effect with VEGF. Moreover, high-grade meningiomas express high levels of COX-2 and VEGF in tumor cells (16, 17). HIF-1α is an extremely potent factor involved in tumor cell growth and proliferation, and it is highly expressed in meningiomas, with a significant positive correlation with recurrence and pathological type (18). One of its mechanisms is that hypoxia-inducible factor stimulates VEGF to form new blood vessels in tumors, promoting tumor growth (19). MMP expression is also associated with the invasive behavior and recurrence of meningiomas (20). Ang2, which is considered a tumor-specific growth factor, is a strong enhancer of sprouting angiogenesis and destabilizes tumor vasculature, which can change the tumor microenvironment and promote tumor growth and metastasis. This effect is fully demonstrated and enhanced in the presence of VEGF (21). Moreover, Ang2 enhances tumor invasiveness and facilitates metastasis by activating and upregulating MMP (22). Consequently, CSDH creates a conducive environment for meningioma occurrence and progression. In this instance, the meningioma fused with the outer membrane of the CSDH, potentially linked to these inflammatory mediators.

The role of prior head trauma in stimulating meningioma development has been previously described in few studies, but remains controversial (23, 24). Traumatic brain injury (TBI) represents a critical worldwide health problem.Especially the elderly patients have a worse mortality and functional outcome,who are also more likely to be on antithrombotic therapy,which is a high-risk factor for chronic subdural hematoma after trauma.And TBI triggered cascades of inflammation (25–28). It is generally believed that chronic inflammatory responses after trauma cause or promote meningioma development (23, 29). From this perspective, CSDH formation after trauma creates a favorable environment for meningioma growth, as previously noted, owing to the high expression of inflammatory factors in both the hematoma and its membrane. Consequently, a meningioma >3 cm was found three years later and closely associated with the outer membrane of the traumatic CSDH. This thus establishes a triad of reactions involving head trauma, chronic subdural hematoma (CSDH), and meningioma.So this may provide new evidence that trauma is a risk factor for meningioma,Cleverly, the CSDH serves as a critical intermediary link in the process. However, in this case, the CSDH was cured clinically and radiologically after burr-hole drainage. Theoretically, the inflammatory factors should be significantly reduced or even disappear after surgery, but a meningioma still grew rapidly in a short period of time and fuse with the membrane. Based on our aforementioned analysis, it is evident that both the hematoma cavity and the outer membrane of CSDH contain a variety of inflammatory mediators. Furthermore, some of these substances play a promoting role in the development and progression of meningiomas. Specifically, they can directly stimulate tumor cell growth and proliferation, induce angiogenesis in tumors, modify the tumor microenvironment, and enhance tumor invasivenes,So could it be considered that the residual outer membrane is still a potential risk factor? The necessity of surgical removal of the outer membrane of CSDH deserves further exploration. In addition, it is widely acknowledged that a small amount of chronic subdural hematoma (CSDH) can be managed conservatively through medication. The primary approach involves administering low-dose oral glucocorticoids and atorvastatin calcium, which serve to suppress the inflammatory response and facilitate hematoma absorption (30).Then, the issue of whether patients who have been cured through surgery also need to use glucocorticoids for a period of time and take atorvastatin calcium for a long time is worth further discussion. Anyway this suggests that if CSDH occurs after trauma, we should be alert to the possibility of meningioma and require regular follow-up.

## 4 Conclusion

We report the first case of a meningioma growing under the outer membrane of a traumatic CSDH after burr-hole drainage. This phenomenon further emphasizes the important role of inflammatory stimulation caused by trauma and chronic subdural hematoma in the development of meningiomas. This may provide a new evidence that head trauma is a risk factor for meningioma.

## Data Availability

The datasets presented in this article are not readily available because of ethical and privacy restrictions. Requests to access the datasets should be directed to the corresponding author/s.
