# Supplementary figures and images for: Meningioma growth beneath the outer membrane of a traumatic chronic subdural hematoma after burr-hole drainage: a case report and literature review
Source: Front Oncol. 2025 May 16;15:1517778. doi: 10.3389/fonc.2025.1517778 (PMC12122768; doi:10.3389/fonc.2025.1517778)

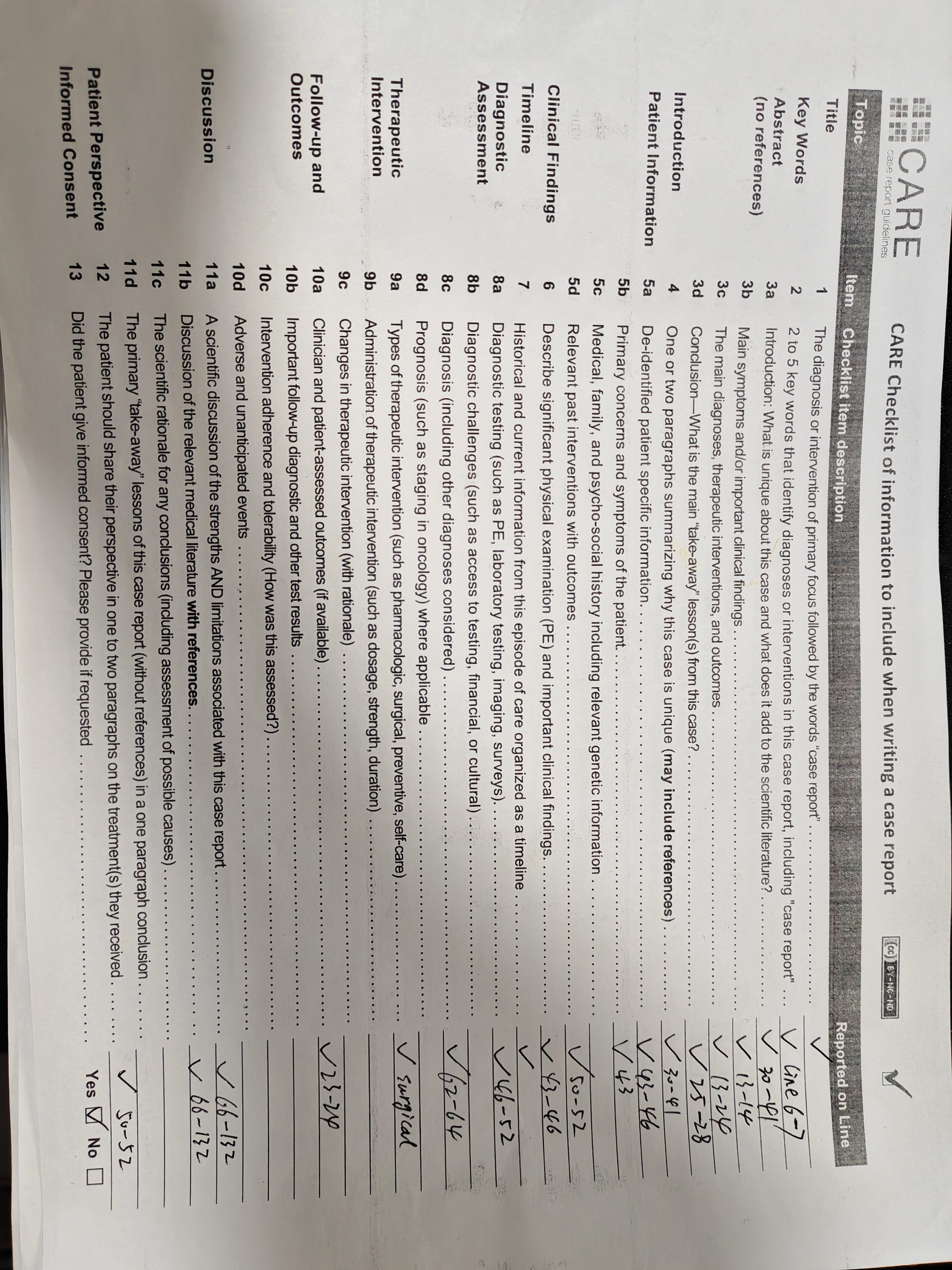

Supplement: Supplementary file 2 [file Image1.jpeg]
