# Supplementary material for: Meningioma growth beneath the outer membrane of a traumatic chronic subdural hematoma after burr-hole drainage: a case report and literature review
Source: Front Oncol. 2025 May 16;15:1517778. doi: 10.3389/fonc.2025.1517778 (PMC12122768; doi:10.3389/fonc.2025.1517778)
Supplement: Supplementary file 1 [file DataSheet1.pdf]

Statement of the Ethics Committee of Zhejiang Hospital

Dear editor:

The case report titled " Meningioma grows beneath the outer membrane of a traumatic chronic subdural hematoma after burr-hole drainage: a case report and literature review" is written by Tao Xiong of our hospital. The case information mentioned in the article has obtained the patient's informed consent and the identifiable information of the case has been hidden. The author has declared that he will fully protect the patient's personal privacy.

The Ethical Review hereby approves the publication of this case report in your journal!

Signature of the chairman/vice chairman:

Ethical Opinion No.: 2024-CA-28

Ethics Committee of Zhejiang Hospital

Oct. 07, 2024

浙江医院 伦理审查委员会

扫描全能王 创建
